# Adsorptive Cathodic Stripping Voltammetric Determination of Cefoperazone in Bulk Powder, Pharmaceutical Dosage Forms, and Human Urine

**DOI:** 10.1155/2013/367914

**Published:** 2013-09-10

**Authors:** Vu Dang Hoang, Dao Thi Huyen, Phan Hong Phuc

**Affiliations:** Department of Analytical Chemistry and Toxicology, Hanoi University of Pharmacy, 13-15 Le Thanh Tong, Hanoi, Vietnam

## Abstract

The electroreduction behaviour and determination of cefoperazone using a hanging mercury drop electrode were investigated. Cyclic voltammograms of cefoperazone recorded in universal Britton-Robinson buffers pH 3–6 exhibited a single irreversible cathodic peak. The process was adsorption-controlled. Britton-Robinson buffer 0.04 M pH 4.0 was selected as a supporting electrolyte for quantitative purposes by differential pulse and square wave adsorptive cathodic stripping voltammetry. The experimental voltammetric conditions were optimized using Central Composite Face design. A reduction wave was seen in the range from −0.7 to −0.8 V. These voltammetric techniques were successfully validated as per ICH guidelines and applied for the determination of cefoperazone in its single and sulbactam containing powders for injection and statistically comparable to USP-HPLC. They were further extended to determine cefoperazone in spiked human urine with no matrix effect.

## 1. Introduction

Cefoperazone is a third-generation cephalosporin antibiotic effective in treating *Pseudomonas aeruginosa* infections, which are otherwise resistant to other antibiotics. Chemically, it is described as (6R,7R)-7-[(2R)-2-{[(4-ethyl-2,3-dioxopiperazin-1-yl)carbonyl]amino}-2-(4-hydroxyphenyl)acetamido]-3-{[(1-methyl-1H-1,2,3,4-tetrazol-5-yl)sulfanyl]methyl}-8-oxo-5-thia-1-azabicyclo[4.2.0]oct-2-ene-2-carboxylic acid. Its good activity, particularly against *Enterobacteriaceae* and *Bacteroides* spp., has been enhanced in the presence of the beta-lactamase inhibitor sulbactam. It is given intramuscularly or intravenously as the sodium salt. Although the drug is mainly excreted in the bile, urinary excretion primarily by glomerular filtration accounts for up to 30% of a dose unchanged within 12 to 24 hours [1].

In the literature, the determination of cefoperazone has been achieved by high performance liquid chromatography (HPLC) [2–6], thin layer chromatography [7], electrophoresis [8], UV spectrophotometry [9–14], spectrofluorimetry [15, 16], colorimetry, and atomic absorption spectrometry [17]. Although voltammetric techniques were also used for this purpose [18–21], the electroanalytical mechanism of cefoperazone was not clearly presented as well as its determination in the presence of sulbactam not being investigated. This work aimed at presenting a validated, simple, and sensitive adsorptive stripping voltammetric procedure for the quantification of cefoperazone in pharmaceutical formulations and spiked human urine, in particular overcoming the above-mentioned drawbacks in voltammetrically studied cefoperazone.

## 2. Experimental

### 2.1. Reagents and Apparatus

Cefoperazone standard was kindly supplied by the National Institute of Drug Quality Control (Vietnam) and used without further purification. A standard stock solution of 500 *μ*g/mL cefoperazone was prepared in water and protected from light in a refrigerator. This solution was stable for at least one week as voltammetrically tested. Working standards of cefoperazone were freshly prepared just before the assay by adding appropriate amounts of stock solution with supporting electrolytes to the mark in 25 mL volumetric flasks. Supporting electrolytes (i.e., acetate pH 4.0, phosphate pH 4.0, and Britton-Robinson universal buffers) were prepared on the day the experiment was performed. Other chemicals were of analytical grade and purchased from Merck (Germany). Urine collected from healthy subjects was used for cefoperazone-spiked samples. Freshly prepared deionized doubly distilled water (Maxima Ultra Pure Water, Elga-Prima Corp, UK) was used throughout this study.

pH measurements were performed on a CyberScan pH 510 (Eutech Instruments Pte Ltd., Singapore). All voltammetric measurements were carried out at 25°C with a 797 VA Computrace (Metrohm AG, Switzerland) in connection with a Dell computer using Microsoft Windows XP and controlled by VA Computrace Software 1.3. The three-electrode system consisted of a hanging mercury drop electrode (HMDE) as working electrode, a platinum wire as auxiliary electrode, and an Ag*|*AgCl*|*KCl(3 M) as reference electrode. A medium size mercury drop was employed. Oxygen-free nitrogen gas was used for the removal of dissolved oxygen from the measured solutions for 100 s with a stirring rate of 2000 rpm. After a preconcentration step, the solutions were left quiescent for 15 s equilibrium before measurement. 

The cyclic voltammetric behaviour of 0.03 *μ*g/mL cefoperazone in the Britton-Robinson universal buffers at the HMDE was studied. The experimental parameters were accumulation potential −0.3 V, accumulation time 100 s, scan rate 0.3 V/s, pulse step 0.005 V, and potential range from −0.3 to −1.2 V. The optimization process started with the selection of buffer type with voltammetric measurements of 0.03 *μ*g/mL cefoperazone. Optimization process was carried out using Modde 9.1 software (Umetrics, Sweden). All experiments were performed in a randomized order as proposed by the software so as to minimize the effect of uncontrolled factors that may introduce a bias on the response. To determine the optimal factors, the optimizer function of Modde software (i.e., Nelder Mead simplex method) was used. Unless stated otherwise, the differential pulse and square wave adsorptive cathodic stripping voltammograms (abbreviated as DP-AdCSV and SW-AdCSV, resp.,) were recorded in triplicate for each programmed run. 

### 2.2. Sample Preparation

A quantity of powders for injection equivalent to 500 mg cefoperazone was accurately weighed and appropriately diluted with water to obtain ca. 2.5 *μ*g/mL cefoperazone test solution.

### 2.3. Analysis of Samples

The general procedure adopted for obtaining adsorptive stripping voltammograms was as follows. For pharmaceutical formulations, 0.3 mL of test solution was diluted with supporting electrolyte solution (i.e., Britton-Robinson universal buffer pH 4.0) to the mark in a 25 mL volumetric flask. For spiked human urine, accurately measured aliquots of 2.5 *μ*g/mL cefoperazone were pipetted into 25 mL volumetric flasks, subsequently added with 0.025 mL human urine, and diluted with supporting electrolyte solution to the mark. The content of these flasks was completely transferred into the voltammetric cell for measurement after 5 min mechanical shaking.

## 3. Results and Discussion

### 3.1. Characteristics of the Electrode Process

**Figure 1:**
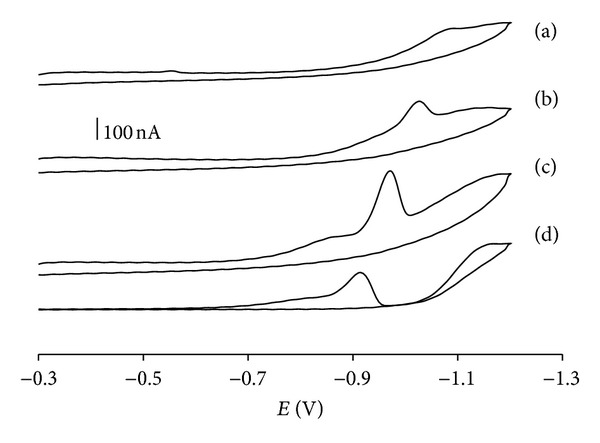
Cyclic voltammograms of 0.03 *μ*g/mL cefoperazone in universal Britton-Robinson buffers pH 6.0 (a), 5.1 (b), 4.0 (c), and 3.0 (d).

A well-defined single peak was yielded in the cathodic direction in the pH range 3–6 (Figure 1). The peak current obtained without accumulation was substantially smaller than that obtained after a preconcentration step (data not shown). It strongly indicates that cefoperazone adsorbs readily onto the surface of HMDE, and a considerable increase in sensitivity can be gained by adsorptive stripping voltammetry. The peak potential (*E*_*p*_) shifted to more negative values and broadened upon the increase of medium pH revealing the involvement of protons in the electrode reaction, and that the proton-transfer reaction precedes the electron transfer [22]. No oxidation peak was observed in the positive scanning half-cycle confirming the irreversible nature of the electrode process. This irreversible cathodic peak disappeared at pH ≤ 2 and ≥7 (data not shown). 

**Scheme 1:**
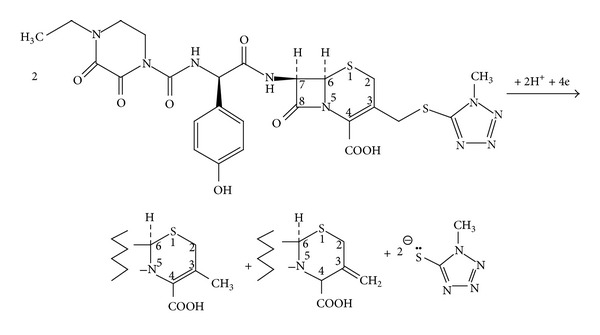
Proposed mechanism of cefoperazone reduction in acidic medium at the mercury electrode.

With reference to most cephalosporins [23, 24], the electrochemical activity of cefoperazone could be attributed to the reduction of the 3-4 double bond of the cephem nucleus activated by the [(1-methyl-1Htetrazol-5-yl)sulphanyl]methyl group present at C_3_ (Scheme 1).

The value of *αn*_*a*_ (product of symmetry transfer coefficient *α* and number of electrons *n*_*a*_ transferred in the rate-determining step) of 0.603 was estimated from slope value of the obtained *E*_*p*_ versus ln⁡*v* plot at pH 4.0 (i.e., *E*_*p*_ = −0.0213ln⁡*v* − 0.9267; *R* ^2^ = 0.961, *n* = 4) using the following equation:

(1) $$\begin{array}{c} \frac{\Delta E_{p}\left(\text{V}\right)}{\Delta \ln v\left(\text{m}{\text{V}\;\text{s}}^{-1}\right)}=\frac{0.02569}{2\alpha n_{a}}. \end{array}$$

For the number of electrons *n*_*a*_ transferred in the rate-determining step for the electroreduction of the double bond of the analyte equals 2, the value of the transfer coefficient *α* (0.301) was estimated. This estimated value indicates the symmetry of the energy barrier in such an irreversible reduction process [25].

The number of protons (*p*) involved in the rate-determining step was consequently estimated to be 0.55 (ca. 1) from the slope value of the *E*_*p*_ versus pH plot (i.e., *E*_*p*_ = −0.0537pH − 0.7592; *R* ^2^ = 0,999, *n* = 4) using the following relation:

(2) $$\begin{array}{c} \frac{\Delta E_{p}\left(\text{V}\right)}{\Delta \text{pH}}=\frac{0.059}{\alpha n_{a}}p. \end{array}$$

Given the fact that two electrons (*n*_*a*_ = 2) and one proton (*m* = 1) are involved in the rate-determining step of the electro-reduction process of a cefoperazone molecule as explained above, the proposed scheme was reasonably justified. This is different from the attribution of cefoperazone irreversible electrode reaction by Hammam and coworkers to the reduction of an electroactive group in the sidechain on C-7 [18]. 

On the other hand, linear plot of *i*_*p*_ versus scan rate *v* was obtained at pH 4.0 with slope value of 884.3 nA · V^−1^ s (*R* ^2^ = 0.999, *n* = 4) revealing that the reduction process of cefoperazone at the HMDE is controlled by adsorption. The slope value (1.022 nA V^−1^ s) of log⁡⁡*i*_*p*_ versus log⁡⁡*v* plot (*R* ^2^ = 0.999, *n* = 4) is very close to the expected theoretical value 1.0 for an ideal reaction of surface species. This indicates again the strong adsorptive behavior of cefoperazone onto the mercury electrode surface [26].

### 3.2. Optimal Parameters and Experimental Conditions

Although DP-AdCSV and SW-AdCSV measurements of 0.03 *μ*g/mL cefoperazone recorded in Britton-Robinson, acetate, phosphate buffers pH 4.0 showed a well-defined sharp peak, the peak most intensified in Britton-Robinson medium. This is in agreement with previously reported data [19]. Accordingly, this type of buffer was chosen as a supporting electrolyte for further investigation on voltammetric parameters, which most influenced the peak current magnitude on preliminary measurements. 

The optimization process was done using a Central Composite Face experiment design with 6 variables as presented in Table 1. While implementing this experiment design, other voltammetric parameters were kept constant. 

**Figure 2:**
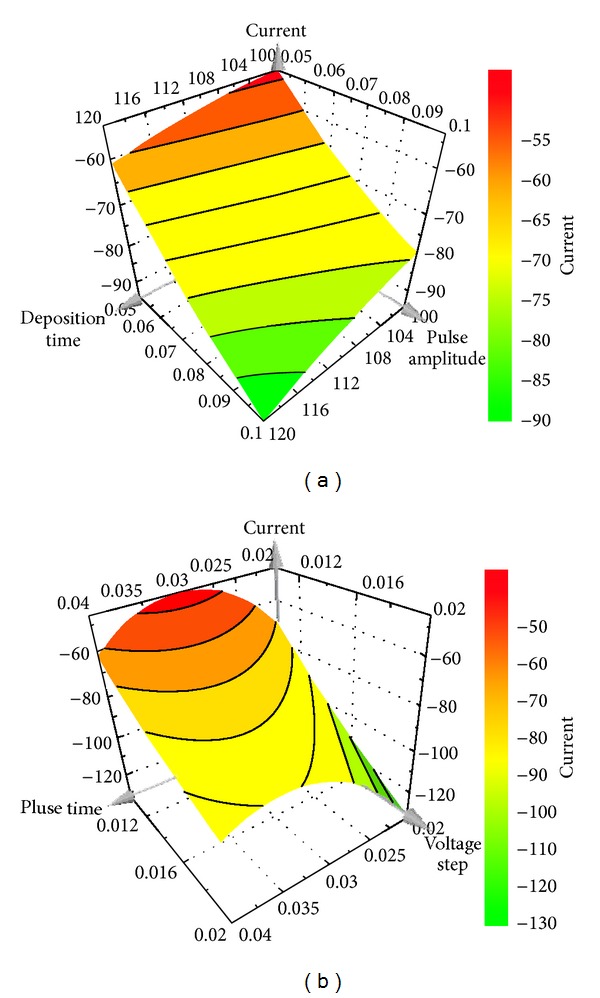
Response surface plots for the influence of deposition time and pulse amplitude (pulse time 0.02 s, voltage step 0.02 V, and voltage step time 0.05 s) (a) and pulse time and voltage step (deposition time 120 s, pulse amplitude 0.1 V, and voltage step time 0.05 s) (b) on DP-AdCSV peak current of 0.03 *μ*g/mL cefoperazone.

**Figure 3:**
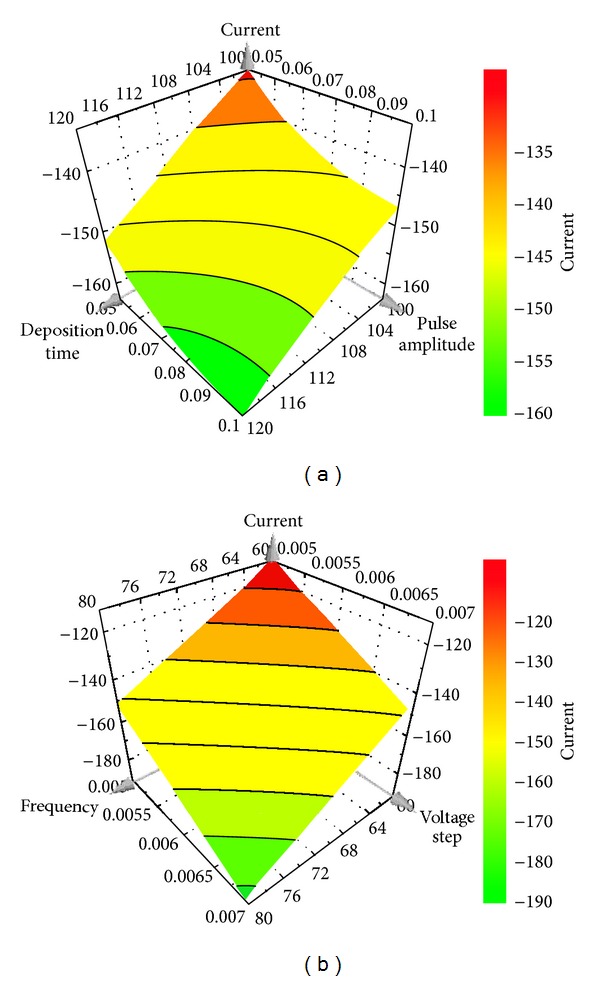
Response surface plots for the influence of deposition time and pulse amplitude (frequency 80 Hz, voltage step 0.007 V) (a) and frequency and voltage step (deposition time 120 s, pulse amplitude 0.1 V) (b) on SW-AdCSV peak current of 0.03 *μ*g/mL cefoperazone.

It is remarked that a minimum peak current of 0.03 *μ*g/mL cefoperazone reached when the values of variables were maximum (i.e., accumulation time (120 s); pulse amplitude (0.1 V); voltage step (0.02 and 0.007 V for DP-AdCSV and SW-AdCSV, resp.); frequency (80 Hz) for SW-AdCSV) and minimum (i.e., pulse time (0.02 s) and voltage step time (0.05 s) for DP-AdCSV) as representatively shown in Figures 2 and 3.

The relationship between peak current and voltammetric variables under study could be mathematically described as follows. According to the *R* ^2^/*Q* ^2^ diagnostic tool (>0.9), the fitted interaction models for both techniques were excellent.

For DP-AdCSV;

(3) I=−5.57u1−23.93u2−7.84u3+16.61u5+0.77u6∗ +5.03u12+5.18u22+3.68u32−18.63u52 −10.91u62−6.23u1u2−9.32u1u5+4.74u1u6 −4.96u2u3+2.91u2u5 +8.37u3u5 (R2=0.984;  Q2=0.999),

and for SW-AdCSV,

(4) I=−8.83u1−20.17u2−5.83u3−20.94u4−2.31u1u4 −2.19u2u3−2.31u3u4 (R2=0.991;  Q2=0.932),

where *u*_1_ is accumulation time; *u*_2_ is voltage step; *u*_3_ is pulse amplitude; *u*_4_ is frequency; *u*_5_ is pulse time; *u*_6_ is voltage step time (*indicates statistically insignificant, *P* > 0.05); *R* ^2^ is goodness of fit; *Q* ^2^: goodness of prediction.

### 3.3. Validation and Application

Under the optimized conditions, the proposed voltammetric techniques (DP-AdCSV and SW-AdCSV) were validated according to ICH guidelines [27].

**Figure 4:**
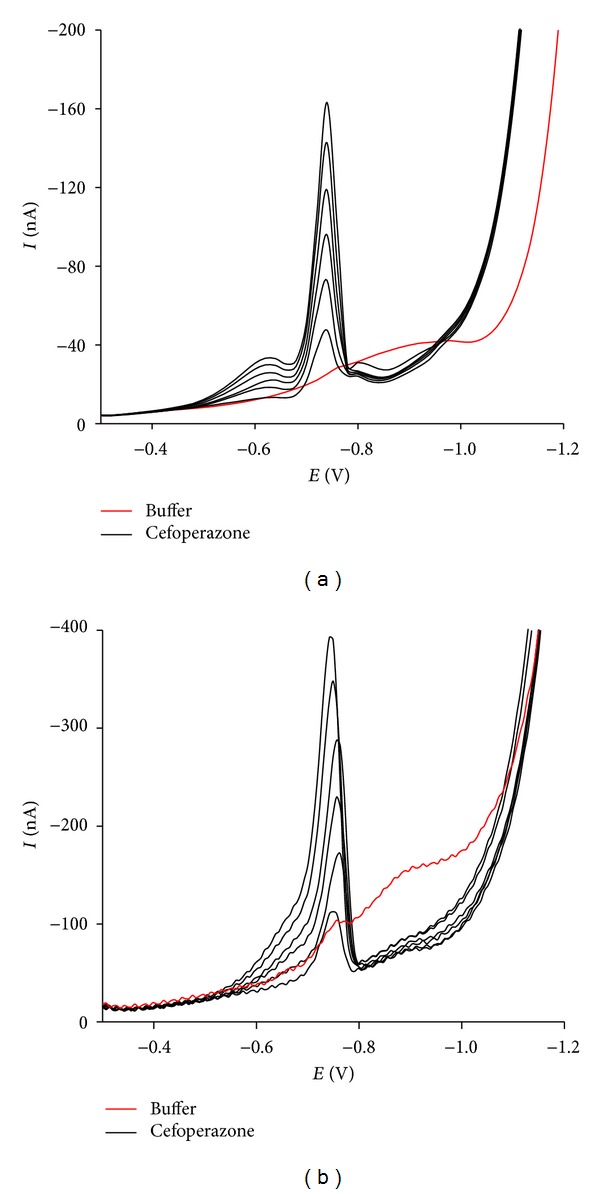
DP-AdCSV (a) and SW-AdCSV (b) measurements of 0.01–0.06 *μ*g/mL cefoperazone in Britton-Robinson buffer pH 4.0.

A linear correlation between the voltammetric peak intensity and drug concentration was obtained over the range 0.01–0.06 *μ*g/mL (Figures 4(a) and 4(b) and Table 2).

The accuracy and intermediate precision of the described stripping voltammetric techniques were examined for 0.01–0.06 *μ*g/mL (Table 3). RSD < 2% and recovery percentages of 99–101% mean that these techniques were accurate and precise. Obviously, the voltammetric determination of cefoperazone was not markedly influenced in the presence of equimolar concentration of sulbactam. In other words, sulbactam does not possess any electrochemical signal in the potential range from −0.3 to −1.2 V.

In our study, USP-HPLC was used as a reference method [28] for quantitatively analyzing cefoperazone in powders for injection. The statistical comparison of voltammetric and liquid chromatographic techniques was done by ANOVA (multiple group mean test) and Bartlett's test for equal variances at a 95% confidence level (Table 4). No statistically significant differences were observed between these chromatographic and voltammetric techniques (*P* > 0.05) suggesting that they had comparable accuracy and precision. In addition, the influence of small variation (5%) of some variables (e.g., buffer pH and concentration, accumulation time) was inspected showing that these voltammetric techniques were fairly robust.

**Figure 5:**
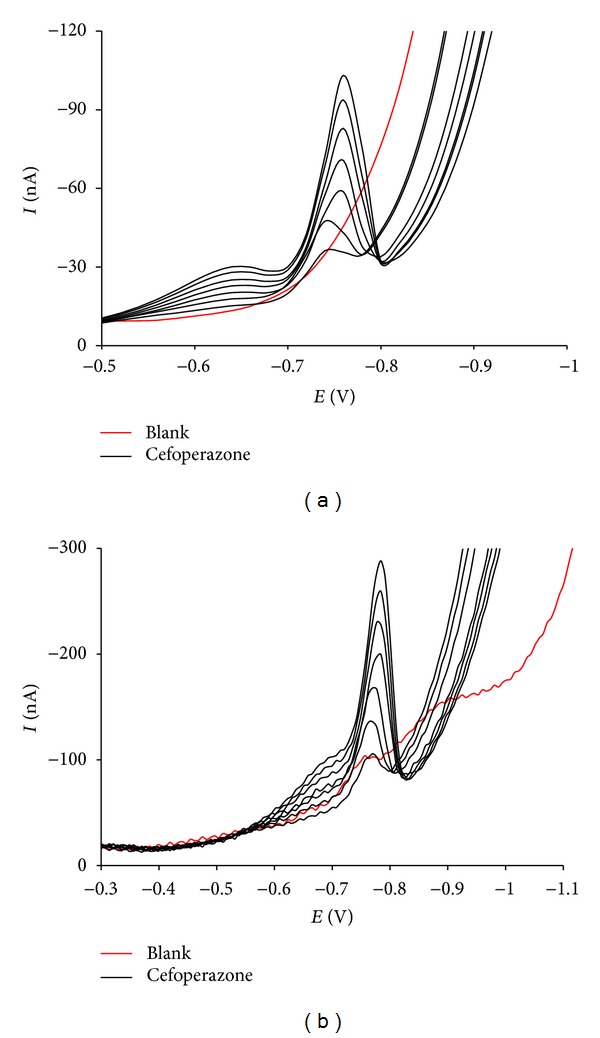
DP-AdCSV (a) and SW-AdCSV (b) measurements of 0.01–0.07 *μ*g/mL cefoperazone in spiked human urine.

For the determination of cefoperazone in spiked human urine, the above-mentioned optimal experimental conditions were also applied. Results show that the analysis was specific as no reduction wave was seen in the range from −0.7 to −0.8 V with pure urine water-diluted by 1000 times (blank) (Figures 5(a) and 5(b)). Supposing that urine levels in excess of 32 *μ*g/mL are maintained for at least 12 h [29], the calibration curve for this analysis was constructed in the corresponding range 0.01–0.07 *μ*g/mL (Table 2). As a consequence of this linear range, the sensitivity (Lower Limit of Quantification (LLOQ)) was 0.01 *μ*g/mL (ca. 1.5 × 10^−8^ M) for both DP-AdCSV and SW-AdCSV techniques. This is comparable to LOQ values reported in other voltammetric studies on cefoperazone [19, 20]. Analyte peak was identifiable, discrete, and reproducible at this concentration (RSD < 20% and recovered within 80–120%). The precision (RSD < 15%) and accuracy (85–115%) were obtained from the back calculation of other calibration standards. It is noteworthy that the urine matrix diminished the peak currents of cefoperazone voltammograms by 10–30% as compared to the corresponding ones in the Britton-Robinson buffer pH 4.0.

This analysis was also intraday- and interday-validated with four spiked quality control (QC) samples (Table 5). The relative analytical recovery (comparing the measured concentration with actual added ones) of these techniques was within the range 85–115% with RSD <15%. The determination of cefoperazone in spiked urine samples was further studied in the presence of sulbactam. According to human pharmacokinetic data, 75% of a 500 mg sulbactam parenteral dose is excreted unchanged in urine over the range 0.015–0.15 *μ*g/mL [30]. The relative recovery of cefoperazone was shown to be 87.9–108.0% for both voltammetric techniques within the sulbactam concentration range specified. Clearly, it is evidenced that DP-AdCSV and SW-AdCSV determination of cefoperazone in human urine satisfactorily meets the requirement for assay methods for biological samples [31].

## 4. Conclusion 

The mechanism of electro-reduction of cefoperazone at the HMDE was elucidated. Using Briton-Robinson buffer 0.04 M pH 4.0 as a supporting electrolyte, DP-AdCSV and SW-AdCSV techniques were developed for the determination of cefoperazone in bulk powder, pharmaceutical dosage forms, and spiked human urine without any prerequisite extraction, separation, and adsorption steps. Voltammetric data were statistically comparable to US pharmacopoeia HPLC meaning that the determination of cefoperazone was accurate and precise. Moreover, the sensitivity and reliability of the determination of cefoperazone in spiked human urine sample were also acceptable. It is recommended that these validated techniques could be used for cefoperazone quality control and pharmacokinetics studies. 

## Figures and Tables

**Table 1:** Variable ranges used in the optimization process of voltammetric measurements of 0.03 *μ*g/mL cefoperazone.

Variable	DP-AdCSV	SW-AdCSV
Accumulation time	100→120 s	
Pulse amplitude	0.05→0.1 V	
Pulse time	0.02→0.04 s	
Frequency		60→80 Hz
Voltage step	0.01→0.02 V	0.005→0.007 V
Voltage step time	0.05→0.06 s	

**Table 2:** Linearity statistical analysis for the proposed voltammetric techniques.

Parameter	Pharmaceutical formulation		Spiked human urine	
	DP-AdCSV	SW-AdCSV	DP-AdCSV	SW-AdCSV
Concentration range (*μ*g/mL)	0.01–0.06	0.01–0.06	0.01–0.07	0.01–0.07
Number of data points: *n*	6	6	7	7
Coefficient of determination: *R* ^2^	0.999	0.999	0.999	0.999
Slope (nA/*μ*g/mL)	−5902	−2338	−3046	−1133
Intercept (nA)	−53	−26	−76	−25
SD of the residuals: *S*_*y*/*x*_	0.8	0.8	1.5	0.9
SD of the slope: *S*_*a*_	20.1	18.1	28.5	17.1
SD of the intercept: *S*_*b*_	0.8	0.7	1.2	0.8

**Table 3:** Accuracy and intermediate precision analysis for the proposed voltammetric techniques.

Number	Cefoperazone 0.03 *µ*g/mL		Cefoperazone + sulbactam (0.03 *µ*g/mL)	
	DP-AdCSV	SW-AdCSV	DP-AdCSV	SW-AdCSV
1	101.4	101.2	99.4	97.3
2	99.5	97.9	101.2	98.4
3	99.1	100.8	101.6	98.9
4	99.6	99.9	100.1	100
5	100.4	100.4	100.3	100.6
6	101.1	98.3	101.2	101.6

Mean	100.2	99.8	100.6	99.5
SD	0.9	1.4	0.8	1.5
RSD (%)	0.9	1.4	0.8	1.5

**Table 4:** Assay results for the determination of cefoperazone in powders for injection by the proposed voltammetric techniques and USP-HPLC.

Pharmaceutical formulation	Recovery ± SD % (*n* = 6)		
	HPLC	DP-AdCSV	SW-AdCSV
	Cefoperazone 500 or 1000 mg*		
Bacamp	99.8 ± 0.7	100.2 ± 0.4	99.9 ± 1.3
Cefobid	100.8 ± 1.3	99.5 ± 0.5	101.2 ± 1.2
Neoaxon*	100.7 ± 0.7	99.2 ± 0.4	100.2 ± 0.5

	Cefoperazone 500 or 1000 mg** + sulbactam 500 or 1000 mg**		
Cefactam**	100.1 ± 1.1	101.1 ± 0.2	102.3 ± 0.4
Sulperazon	100.4 ± 0.9	101.0 ± 0.2	101.1 ± 1.2
Jincetam	100.9 ± 1.1	101.7 ± 0.3	100.8 ± 1.1

**Table 5:** Accuracy and precision data for the determination of cefoperazone in spiked human urine by the proposed voltammetric techniques (*n* = 3).

	Actual conc. (*µ*g/mL)	Experimental conc. (mean ± SD *µ*g/mL)	Precision as RSD (%)	Accuracy (%)
	DP-AdCSV			
Intraday	0.01	0.0097 ± 0.0001	1.0	97.2
	0.03	0.0294 ± 0.0003	1.0	97.9
	0.04	0.0405 ± 0.0002	0.5	101.2
	0.06	0.0605 ± 0.0005	0.8	100. 9
Interday	0.01	0.0092 ± 0.0003	3.3	92.3
	0.03	0.0302 ± 0.0008	2.6	100.7
	0.04	0.0408 ± 0.0001	2.7	102.1
	0.06	0.0600 ± 0.0003	0.5	100.2

	SW-AdCSV			
Intraday	0.01	0.0095 ± 0.0002	2.1	94.8
	0.03	0.0305 ± 0.0005	1.6	101.6
	0.04	0.0408 ± 0.0002	0.5	102.0
	0.06	0.0607 ± 0.0011	1.8	101.0
Interday	0.01	0.0097 ± 0.0002	2.1	96.7
	0.03	0.0303 ± 0.0002	0.7	101.1
	0.04	0.0405 ± 0.0003	0.7	101.3
	0.06	0.0602 ± 0.0005	0.8	100.3

## References

[1] Sean CS (2011). *Martindale: The Complete Drug Reference. Cefoperazone Monograph*.

[2] Gopinath R, Rajan S, Meyyanathan SN, Krishnaveni N, Suresh B (2007). A RP-HPLC method for simultaneous estimation of paracetamol and aceclofenac in tablets. *Indian Journal of Pharmaceutical Sciences*.

[3] Tsou T-L, Huang Y-C, Lee C-W, Lee A-R, Wang H-J, Chen S-H (2007). Simultaneous determination of ampicillin, cefoperazone, and sulbactam in pharmaceutical formulations by HPLC with *β*-cyclodextrin stationary phase. *Journal of Separation Science*.

[4] El-Shanawani AA (1998). HPLC determination of sulbactam, sultamicillin tosylate, cefaclor, ampicillin and cefoperazone in pharmaceutical preparations. *Acta Poloniae Pharmaceutica*.

[5] Elkady EF, Abbas SS (2011). Development and validation of a reversed-phase column liquid chromatographic method for the determination of five cephalosporins in pharmaceutical preparations. *Journal of AOAC International*.

[6] Selavka CM, Krull IS, Bratin K (1986). Analysis for penicillins and cefoperazone by HPLC-photolysis-electrochemical detection (HPLC-hv-EC). *Journal of Pharmaceutical and Biomedical Analysis*.

[7] Mohamed FA, Saleh GA, El-Shaboury SR, Rageh AH (2008). Selective densitometric analysis of cephalosporins using dragendorff’s reagent. *Chromatographia*.

[8] Pajchel G, Tyski S (2000). Adaptation of capillary electrophoresis to the determination of selected cephalosporins for injection. *Journal of Chromatography A*.

[9] Senthilraja M, Sanjaypai P (2006). Spectrophotometric method for the determination of cefoperazone sodium in pharmaceutical formulations. *Indian Journal of Pharmaceutical Sciences*.

[10] Jane J, Subrahmanyam EVS, Sathyanarayana D (2006). Spectrophotometric determination of certain cephalosporins. *Asian Journal of Chemistry*.

[11] Salem H (2004). Selective spectrophotometric determination of phenolic *β*-lactam antibiotics in pure forms and in their pharmaceutical formulations. *Analytica Chimica Acta*.

[12] Saleh GA, Askal HF, Radwan MF, Omar MA (2001). Use of charge-transfer complexation in the spectrophotometric analysis of certain cephalosporins. *Talanta*.

[13] Saleh GA, El-Shaboury SR, Mohamed FA, Rageh AH (2009). Kinetic spectrophotometric determination of certain cephalosporins using oxidized quercetin reagent. *Spectrochimica Acta A*.

[14] Parra A, Garcia-Villanova J, Rodenas V, Gomez MD (1994). First and second derivative spectrophotometric determination of cefoperazone and sulbactam in injections. *Journal of Pharmaceutical and Biomedical Analysis*.

[15] El Walily AFM, Abdel-Kader Gazy A, Belal SF, Khamis EF (1999). Selective spectrofluorimetric determination of phenolic *β*-lactam antibiotics through the formation of their coumarin derivatives. *Journal of Pharmaceutical and Biomedical Analysis*.

[16] Bebawy LI, El Kelani K, Fattah LA (2003). Fluorimetric determination of some antibiotics in raw material and dosage forms through ternary complex formation with terbium (Tb3+). *Journal of Pharmaceutical and Biomedical Analysis*.

[17] Salem H, Askal H (2002). Colourimetric and AAS determination of cephalosporins using Reineck’s salt. *Journal of Pharmaceutical and Biomedical Analysis*.

[18] Hammam E, El-Attar MA, Beltagi AM (2006). Voltammetric studies on the antibiotic drug cefoperazone. Quantification and pharmacokinetic studies. *Journal of Pharmaceutical and Biomedical Analysis*.

[19] Billová S, Kizek R, Jelen F, Novotná P (2003). Square-wave voltammetric determination of cefoperazone in a bacterial culture, pharmaceutical drug, milk, and urine. *Analytical and Bioanalytical Chemistry*.

[20] Ali AM, El-Maali NA, Ghandour MA (1993). Determination of cefobid using adsorptive stripping voltammetric technique in different media. *Electroanalysis*.

[21] Dogan B, Golcu A, Dolaz M, Ozkan SA (2009). Electrochemical behaviour of the bactericidal cefoperazone and its selective voltammetric determination in pharmaceutical dosage forms and human serum. *Current Pharmaceutical Analysis*.

[22] Zuman P (1969). *The Elucidation of Organic Electrode Processes*.

[23] Ogorevc B, Gomiscek S (1991). Electrochemical analysis of cephalosporin antibiotics. *Journal of Pharmaceutical and Biomedical Analysis*.

[24] Zuman P, Kapetanovic V, Aleksic M (2000). Recent developments in electroanalytical chemistry of cephalosporins and cefamycins. *Analytical Letters*.

[25] Nicholson RS (1965). Theory and application of cyclic voltammetry for measurement of electrode reaction kinetics. *Analytical Chemistry*.

[26] Laviron E, Roullier L, Degrand C (1980). A multilayer model for the study of space distributed redox modified electrodes. Part II. Theory and application of linear potential sweep voltammetry for a simple reaction. *Journal of Electroanalytical Chemistry*.

[27] Baber N (1994). International conference on harmonisation of technical requirements for registration of pharmaceuticals for human use (ICH). *British Journal of Clinical Pharmacology*.

[28] USP30-NF25

[29] Craig WA, Gerber AU (1981). Pharmacokinetics of cefoperazone: a review. *Drugs*.

[30] Foulds G, Stankewich JP, Marshall DC (1983). Pharmacokinetics of sulbactam in humans. *Antimicrobial Agents and Chemotherapy*.

[31] Niazi SK (2007). *Handbook of Bioequivalence Testing*.

